# Comparison of Antimicrobial Sensitivity to Older and Newer Quinolones versus Piperacillin-Tazobactam, Cefepime and Meropenem in Febrile Patients with Cancer in two Referral Pediatric Centers in Tehran, Iran

**DOI:** 10.4084/MJHID.2014.045

**Published:** 2014-07-01

**Authors:** AR. Nateghian, JL. Robinson, P. Vosough, M. Navidinia, M. Malekan, A. Mehrvar, B. Sobouti, P. Bahadoran, Z. Gholinejad

**Affiliations:** 1 Department of Pediatrics, Iran University of Medical Sciences, Tehran, Iran; 2 Department of Pediatrics, University of Alberta and Stollery Children’s Hospital. Edmonton, Canada; 3 Mahak Hospital and Rehabilitation Complex, Tehran, Iran; 4 Pediatric Infections Research Center, Mofid Children Hospital, Shahid Beheshti University of Medical Sciences, Tehran, Iran; 5 Aliasghar Children’s Hospital, Iran University of Medical Sciences, Tehran, Iran

## Abstract

**Background:**

Infection in pediatric cancer patients has become a concerning problem due to increasing antimicrobial resistance. The goal of this study was to determine the antimicrobial resistance patterns of blood isolates from pediatric oncology patients in Iran to determine if there was significant resistance to quinolones

**Methods:**

Children with cancer who were admitted with or developed fever during admission to Aliasghar Children’s Hospital or Mahak Hospitals July 2009 through June 2011 were eligible for enrollment. Two blood cultures were obtained. Antimicrobial sensitivity test was performed for ciprofloxacin, moxifloxacin, gatifloxacin, meropenem, cefepime, and piperacillin-tazobactam on isolates from children who were bacteremic.

**Results:**

Blood cultures were positive for 38 episodes in 169 enrolled children but 9 episodes were excluded as blood cultures were thought to be contaminated, yielding a bacteremia rate of 29/160 (18%). The mean age of children and the stage of malignancy did not differ between those with and without bacteremia. Meropenem was the most likely antibiotic to cover isolates (97%) with cefepime having the lowest coverage rate (21%). Quinolone coverage ranged from 63% to 76%.

**Conclusion:**

Quinolones may not be suitable for use as empiric therapy in febrile pediatric oncology patients in Iran.

## Introduction

Infectious diseases are common life threatening complications in patients with cancer, resulting in repeated admissions with considerable interruption of treatment for the underlying malignancy.^1^ Although the mortality rate for infectious diseases has been decreasing over recent decades, most children with febrile neutropenia are treated as inpatients with long durations of admission.^2^

Recently, there are reports of successful outpatient management of febrile neutropenia in children with malignancies with oral antibiotics (typically quinolones).^3^ However, there is a concerning increase in the rate of antimicrobial resistance both in developed and developing countries, including quinolone resistance in *Escherichia coli* and *Klebsiella pneumoniae*. Therefore, the predicted efficacy of quinolones for invasive bacterial infections in children with malignancies can be informative as these drugs are used in this setting despite not yet being approved in children.^3^

Aliasghar Children’s Hospital (an educational hospital affiliated with the Tehran University of Medical Sciences) and Mahak Hospital (a subspecialty non-profit center for children with cancer) are referral pediatric oncology centers in Tehran. Almost all febrile pediatric patients with cancer (with or without neutropenia) are admitted rather than treated as outpatients as there is no consensus on when and how to use risk assessments for selecting patients for outpatient management.^4^

The primary objective of this study was to determine the in-vitro susceptibility for blood culture isolates in these hospitals to older and newer quinolones and to compare this with the susceptibilities to current candidates for intravenous monotherapy in this population.

## Materials and Methods

All children with any cancer irrespective of their stage of disease (including those who had finished chemotherapy or relapsed) who were admitted with fever or developed fever in Aliasghar Children’s Hospital or Mahak Hospitals from July 2009 through June 2011 were eligible for enrollment in this descriptive prospective study. The same child could be enrolled more than once in the study. Informed consent was obtained from parents.

Fever was defined as a single episode of temperature above 38.5 degrees C or at least two episodes of 38 degrees C one hour apart. For each febrile patient, two blood cultures were collected simultaneously before starting or changing the antibiotic therapy. Of these blood cultures, one was sent to the hospital laboratory and processed by the standard procedure and the other was sent to BACTEC the Pediatric Infectious Diseases Research Center located in Mofid Children’s Hospital and processed by BACTEC technology to improve the sensitivity. Standard methods were used for identification of organisms. Children who had positive blood cultures for common skin contaminants were considered to be bacteremic only if both samples were positive for the same microorganism. Antimicrobial sensitivity test was performed for ciprofloxacin, moxifloxacin, gatifloxacin, meropenem, cefepime, and piperacillin-tazobactam according to Clinical and Laboratory Standards Institute (CLSI) methods using the standard disk diffusion method from MAST Co (Mast Co, Merseyside, UK). A questionnaire was completed for each patient on admission and data was analyzed by SPSS software version-16 using appropriate statistical methods.

Data are presented as numbers and proportions. Continuous variables are presented as mean and standard deviation.

## Results

There were 173 febrile episodes during the period of study. Parental consent was not obtained for 4 of these episodes. Blood cultures were positive for 38 of the remaining 169 episodes but 9 episodes were excluded from further analysis as only a single blood culture was positive for a common skin contaminant, yielding a proven bacteremia rate of 29/160 ([18%). All 9 suspected contaminated blood cultures were from BACTEC alone. For the 29 episodes of bacteremia, both blood cultures were positive in 14 episodes and BACTEC alone in 15 episodes. Ninety-one of these 160 cases were male (57%). The mean age of enrolled patients was 82 months (SD=54.4). The mean age of cases with bacteremia was 71.5 months (SD=55.6) and for those with negative blood cultures or contaminated blood cultures was 84.3 months (SD=57.8) (P value =0.27).

Three enrolled children had two episodes of bacteremia during different admissions. These were not thought to be relapses or recurrences as two of the children had different organisms each time and the third child had the same organism but with different susceptibilities.

The 160 children had 19 different types of malignancies (Table 1). The types of malignancies in enrolled patients with and without bacteremia are shown in Table 1 (P value=0.002). Bacteremia with fever was especially common with acute myeloid leukemia (6 of 16 episodes; 37%). There was no significant difference in stage of malignancy between those with and without bacteremia (P value=0.14).

The organisms causing bacteremia are shown in Table 2 with coagulase negative staphylococci causing 10 of the 29 cases (34%). The coverage rate was highest for meropenem (97%) with cefepime having the lowest coverage rate (21%). Overall susceptibility rates for the three quinolones were very similar to each other, ranging from 63% to 76% (Table 2).

## Discussion

Meropenem had the highest rate of in-vitro coverage for organisms isolated from bacteremic children with malignancies in Iran. In contrast, cefepime susceptibility was only about 21%, a dramatic change from a previous study in the same population hospitals in which susceptibility was over 80%.^5^ Only about 70% of the isolated organisms were susceptible to quinolones with the sample size being too small to compare ciprofloxacin to newer quinolones. Vital gaps in quinolone coverage included *Staphylococcus aureus* and *Klebsiella* species.

In a 2003 study in adults in the United States with malignancies, moxifloxacin had good coverage for gram negative organisms except for *Pseudomonas* species for which it was inferior to ciprofloxacin and levofloxacin.^6^ In total, 78% of gram positive and 64% of gram negatives were susceptible to moxifloxacin. The same group published a study in 2006, showing that gatifloxacin offered good coverage for all but methicillin-resistant *S. aureus*, and *Pseudomonas* and *Acinetobacter* species.^7^ In total, 83% of gram positive and 64% of gram negatives were susceptible to gatifloxacin.^8^

International guidelines for management of children with malignancies and febrile neutropenia were published in 2012.^9^ They describe six validated schemes for identifying children at low risk for serious infection with it not being clear which scheme functions best. The guidelines report that oral antibiotics (often quinolones) appear to be equivalent to intravenous antibiotics for empiric therapy in low-risk children. In a recent meta-analysis specifically on the role of quinolones in children with febrile neutropenia, 10 studies (740 episodes of febrile neutropenia, all considered to be low-risk for poor outcomes) were included.^3^ Using varying definitions, treatment success was obtained in 83% of those on quinolone monotherapy with there being no infection-related mortality. This success rate appeared to be higher than that obtained with the usual intravenous options. However, it is important to recognize that these studies were performed in an era when quinolone resistance was less common than in the current study. In the absence of outcome data, it is difficult to predict what would have happened had all low risk patients in the current study been started on quinolones, but the high rate of in-vitro resistance is concerning.

The major limitation of the current study is that only in-vitro data rather than clinical outcome data was available. Clinical and Laboratory Standards Institute standards do not yet exist for all antibiotics in this study for some of the bacteria isolated. Patients did not have to be neutropenic to be enrolled, and those who were neutropenic were not further classified as low-risk versus high-risk for poor outcomes. It can be difficult to distinguish bacteremia from contaminated blood cultures. Processing one blood culture by BACTEC technology appeared to increase both the number of contaminated blood cultures and the number of true positives.

## Conclusions

Although quinolones appeared to be a good option for children with febrile neutropenia in other countries, one would want to be cautious given the apparent high rate of resistance in Iran. Patients who are bacteremic with pathogens of low virulence such as coagulase negative staphylococci or enterococcus are likely to survive even if empiric antibiotics do not cover that organism, but the same is not true for *E. coli* or *Klebsiella* species, both of which demonstrated significant quinolone resistance is the current study. Given the rapid emergence of multi-resistant gram negatives, it is imperative that rates of quinolone resistance be followed closely in oncology centers where febrile neutropenia is being managed with this class of drug.

## Figures and Tables

**Table 1:** Presence or absence of bacteremia in 160 episodes of fever in children with different malignancies

Malignancy	Group		Total
	Bacteremia	No bacteremia	
ALL	14 (17%)	69 (83%)	83
AML	6(37%)	10(63%)	16
Neuroblastoma	1(33%)	2(67%)	3
Non Hodgkin’s lymphoma	1(50%)	1(50%)	2
Teratoma	1(100%)	0	1
Burkitt’s lymphoma	1(25%)	3(75%)	4
PNET	1(25%)	3(75%)	4
Wilm’s tumor	2(29%)	5(71%)	7
Glioma	1(50%)	1(50)	2
Osteosarcoma	0	1(100%)	1
Retinoblastoma	1(13%)	7(87%)	8
Rhabdomyosarcoma	0	12(100%)	12
Germ cell tumor	0	3(100%)	3
Hodgkin’s lymphoma	0	4(100%)	4
Histiocytosis	0	1(100%)	1
Medulloblastoma	0	5(100%)	5
Renal cell carcinoma	0	1(100%)	1
Ewing’s sarcoma	0	2(100%)	2
Nasopharyngeal carcinoma	0	1(100%)	1
**Total**	**29(18%)**	**131(82%)**	**160**

AML: acute myelogenous leukemia; ALL: acute lymphoblastic leukemia; PNET: primitive neuroectodermal tumor.

**Table 2:** Susceptibility rates for organisms causing bacteremia in children with malignancies (N=29)(%)

Organism	ciprofloxacin	moxifloxacin	gatifloxacin	meropenem	cefepime	piperacillin- tazobactam

*Streptococcus pneumoniae*^3^	NA	3 (100%)	3 (100%)	3 (100%)	2 (67%)	NA
Coagulase negative staphylococcus^10^	8 (80%)	8 (80%)	9 (90%)	10 (100%)	2 (20%)	9 (90%)
*Escherichia coli*^4^	3 (75%)	NA	3 (75%)	4 (100%)	1 (25%)	4 (100%)
*Staphylococcus.aureus*^3^	1 (33%)	1 (33%)	1 (33%)	3 (100%)	1 (33%)	1 (33%)
*Pseudomonas aeruginosa*^1^	1 (100%)	1 (100%)	1 (100%)	1 (100%)	0	0
*Citrobacter freundii*^1^	1 (100%)	NA	1 (100%)	1 (100%)	0	1 (100%)
Streptococcus viridans group^2^	NA	2 (100%)	2 (100%)	2 (100%)	0	2 (100%)
*Enterococcus.faecalis*^1^	0	0	0	1 (100%)	0	1 (100%)
*Enterobacter aerogenes*^1^	1 (100%)	NA	1 (100%)	1 (100%)	0	1 (100%)
*Klebsiella pneumoniae*^3^	0	NA	1 (33%)	2 (67%)	0	1 (33%)

**Total susceptibility**	**15/24 (63%)**	**15/20 (75%)**	**22/29 (76%)**	**28/29 (97%)**	**6/29 (21%)**	**20/26 (77%)**

NA - not available as break-point not established by Clinical and Laboratory Standards Institute (CLSI)
